# Migrant and refugee populations: a public health and policy perspective on a continuing global crisis

**DOI:** 10.1186/s13756-018-0403-4

**Published:** 2018-09-20

**Authors:** Mohamed Abbas, Tammam Aloudat, Javier Bartolomei, Manuel Carballo, Sophie Durieux-Paillard, Laure Gabus, Alexandra Jablonka, Yves Jackson, Kanokporn Kaojaroen, Daniel Koch, Esperanza Martinez, Marc Mendelson, Roumyana Petrova-Benedict, Sotirios Tsiodras, Derek Christie, Mirko Saam, Sally Hargreaves, Didier Pittet

**Affiliations:** 1 Infection Control Programme and WHO Collaborating Centre on Patient Safety, Faculty of Medicine, University of Geneva Hospitals, Geneva, Switzerland; 2 Médecins sans Frontières, Geneva, Switzerland; 3 CAPPI Servette, Department of Mental Health and Psychiatry, Geneva University Hospitals, Genève, Switzerland; 4 International Centre for Migration, Health and Development, Geneva, Switzerland; 5 Programme Santé Migrants, Department of Community Medicine, Primary Care and Emergency Medicine, Geneva University Hospitals, Geneva, Switzerland; 6 Geneva, Switzerland; 7 Department of Clinical Immunology and Rheumatology, Hannover Medical School, Hannover, Germany; 8 German Center for Infection Research (DZIF), PARTNER Site Hannover-Braunschweig, Hannover, Germany; 9 Division of Primary Care Medicine, Geneva University Hospitals, Geneva, Switzerland; 10 Institute of Global Health, Geneva University, Geneva, Switzerland; 11 Department of Service Delivery & Safety, World Health Organization, Geneva, Switzerland; 12 Division of Communicable Diseases, Federal Office of Public Health, Bern, Switzerland; 13 Health Unit, International Committee of the Red Cross (ICRC), Geneva, Switzerland; 14 Division of Infectious Diseases & HIV Medicine, Department of Medicine, Groote Schuur Hospital, University of Cape Town, Cape Town, South Africa; 15 International Organization for Migration (IOM), Migration Health Division (MHD), Regional office (RO), Brussels, Belgium; 16 4th Department of Medicine, Medical School, National and Kapodistrian University of Athens, Athens, Greece; 17 Hellenic Centre for Disease Control & Prevention, Athens, Greece; 18 Division of environmental health, Institute of Global Health, Faculty of Medicine, University of Geneva, Geneva, Switzerland; 19 Communication in Science, Geneva, Switzerland; 20 Section of Infectious Diseases and Immunity, Department of Medicine, Imperial College London, Hammersmith Hospital, London, W12 0HS UK; 21 The Institute for Infection and Immunity, St George’s, University of London, London, WC1E 7HU UK

**Keywords:** Migrant populations, Refugees, Crisis, Global health, Public health policy, Infectious diseases

## Abstract

The 2015–2017 global migratory crisis saw unprecedented numbers of people on the move and tremendous diversity in terms of age, gender and medical requirements. This article focuses on key emerging public health issues around migrant populations and their interactions with host populations. Basic needs and rights of migrants and refugees are not always respected in regard to article 25 of the Universal Declaration of Human Rights and article 23 of the Refugee Convention. These are populations with varying degrees of vulnerability and needs in terms of protection, security, rights, and access to healthcare. Their health status, initially conditioned by the situation at the point of origin, is often jeopardised by adverse conditions along migratory paths and in intermediate and final destination countries. Due to their condition, forcibly displaced migrants and refugees face a triple burden of non-communicable diseases, infectious diseases, and mental health issues. There are specific challenges regarding chronic infectious and neglected tropical diseases, for which awareness in host countries is imperative. Health risks in terms of susceptibility to, and dissemination of, infectious diseases are not unidirectional. The response, including the humanitarian effort, whose aim is to guarantee access to basic needs (food, water and sanitation, healthcare), is gripped with numerous challenges. Evaluation of current policy shows insufficiency regarding the provision of basic needs to migrant populations, even in the countries that do the most. Governments around the world need to rise to the occasion and adopt policies that guarantee universal health coverage, for migrants and refugees, as well as host populations, in accordance with the UN Sustainable Development Goals. An expert consultation was carried out in the form of a pre-conference workshop during the 4th International Conference on Prevention and Infection Control (ICPIC) in Geneva, Switzerland, on 20 June 2017, the United Nations World Refugee Day.

## Background

**Fig. 1:**
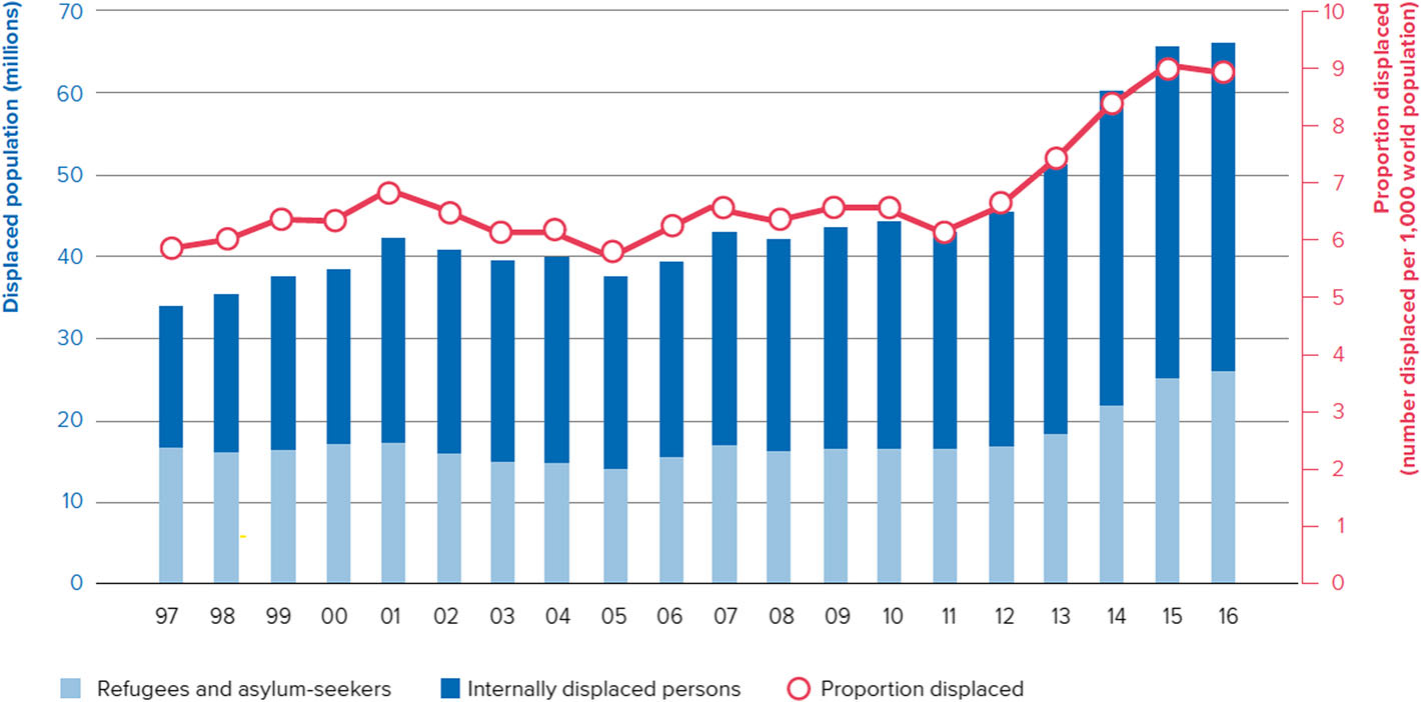
Estimates (in millions) of the global numbers of migrants 1990–2016. © UNHCR, reproduced with permission

The current global refugee crisis peaked in 2015–2016, and by late 2017 the number of people attempting to cross borders globally – although still high – was receding. The highest levels of forced displacement since World War II were observed in 2015**,** with a dramatic increase in the numbers of refugees, asylum-seekers and internally displaced people (IDPs) across the world – from Africa to the Middle East and South Asia. “Desperate” migration towards Europe became increasingly seaborne – with over one million migrants arriving by boat in Greece and Italy in 2015. Such operations are highly risky; in the Mediterranean, several thousand migrants have drowned every year since 2014. At the global level, at least 60′000 migrants have died or gone missing over the past 20 years [1]. Although statistics on migration are difficult to collect, it is necessary to avail oneself of the available data which should be viewed as estimates (Fig. 1) [2].

For purposes of ease of reading, and because we believe that the current nomenclature is arbitrary, in this paper we will use the International Organization for Migration’s (IOM) definition, and refer to a migrant as “any person who is moving or has moved across an international border or within a State away from his/her habitual place of residence, regardless of (1) the person’s legal status; (2) whether the movement is voluntary or involuntary; (3) what the causes for the movement are; or (4) what the length of the stay is.” [3] We will use the term interchangeably with the words refugees and asylum-seekers, whether documented or not.

A hallmark of global migration patterns is that IDPs are the highest in number, and low and middle-income countries host most of the world’s refugees. In 2016, Turkey had more than 3 million refugees and asylum-seekers on its soil, including 2.7 million Syrians; Lebanon had the highest number relative to its population, nearly one in five inhabitants is a refugee. Of this, the United Nations (UN) Secretary General, António Guterres, said “It is so inspiring to see countries with the least often doing the most for refugees” [4]. In this context, the so-called European “migrant crisis” pales in comparison, and perhaps a change in perspective is required, as in Natalie Nougayrède’s words:

“If there was a crisis in 2015, it had less to do with the refugees – who knew what they were fleeing and where they wanted to go – and much more to do [*sic*] with European governments and societies who did not all step up to the plate. In fact, Europe isn’t confronted with a refugee and migrant crisis. It’s the refugees and migrants who are confronted with a crisis of Europe. The scandal is that, in the Mediterranean, they have been paying with their lives.” [5]

The reasons for forced migration and displacement are increasingly varied, but stem from fragility of states, due to armed conflict and civil unrest, extreme poverty, crime, persecution (including political discrimination), failure of governance, or climate change [6–8]. Over half of the refugees globally come from three countries: Syria, Afghanistan and Somalia; yet refugees are the tip of the iceberg, when one considers the number of IDPs. Altogether, more than 1.5 billion people live in the 56 fragile states that engender refugees [9].

On the occasion of the UN World Refugee Day, 20 June 2017, we conducted an expert consultation in the form of a pre-conference workshop during the 4th International Conference on Prevention and Infection Control (ICPIC) in Geneva.

### Basic needs and rights

The 1951 Refugee Convention guarantees basic rights such as liberty, security, right to family life, protection, and freedom of movement [10]. Refugees are therefore not to be returned to their home country against their will. Other basic needs include the right to education and justice. Also, article 23 of the Refugee Convention guarantees the right of refugees to public relief, that is, to access physical and mental health services at the same level as other residents. This fundamental right is also guaranteed by article 25 of the Universal Declaration of Human Rights [11].

In the present crisis, these basic rights have not always been met. While there have been some advances in the form of migrant-friendly hospitals, health systems overall are not sufficiently responsive to migrants, to diversity, or to specific medical and psychosocial care. A needs-based approach is required to address these issues.

**Fig. 2:**
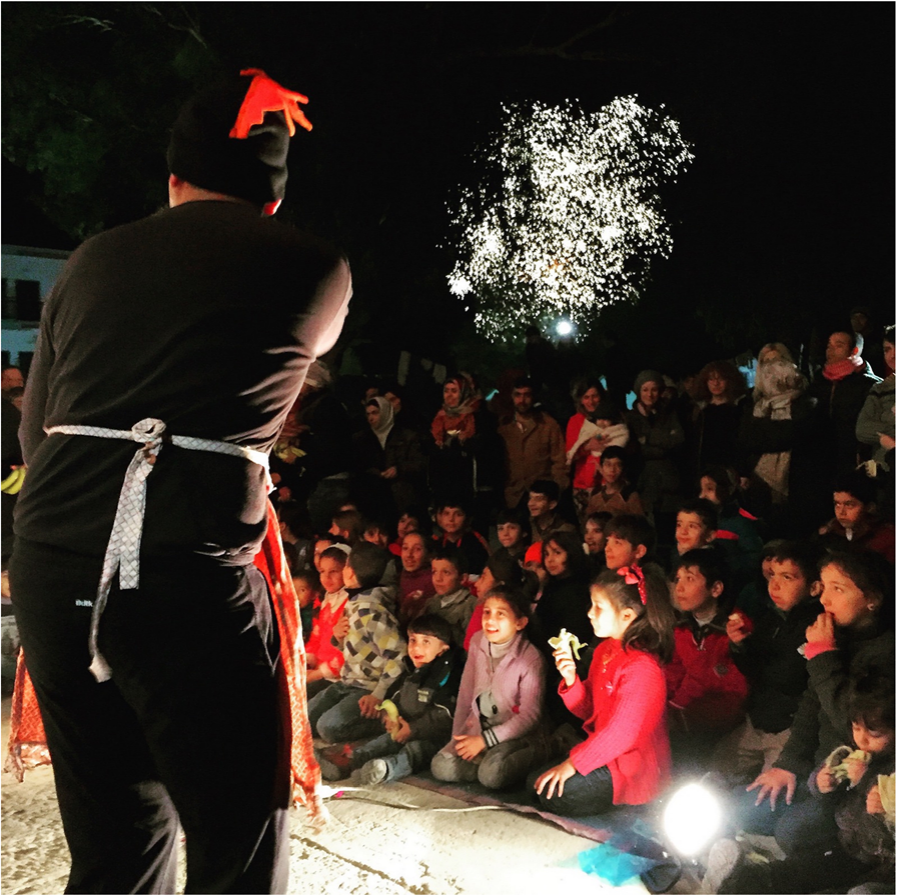
A Greek theatre company gives a show in a refugee camp in Leros. The play, in Greek, is about a little black fish lost in the ocean. None of the spectators understand, but everyone is laughing. © Laure Gabus

**Fig. 3:**
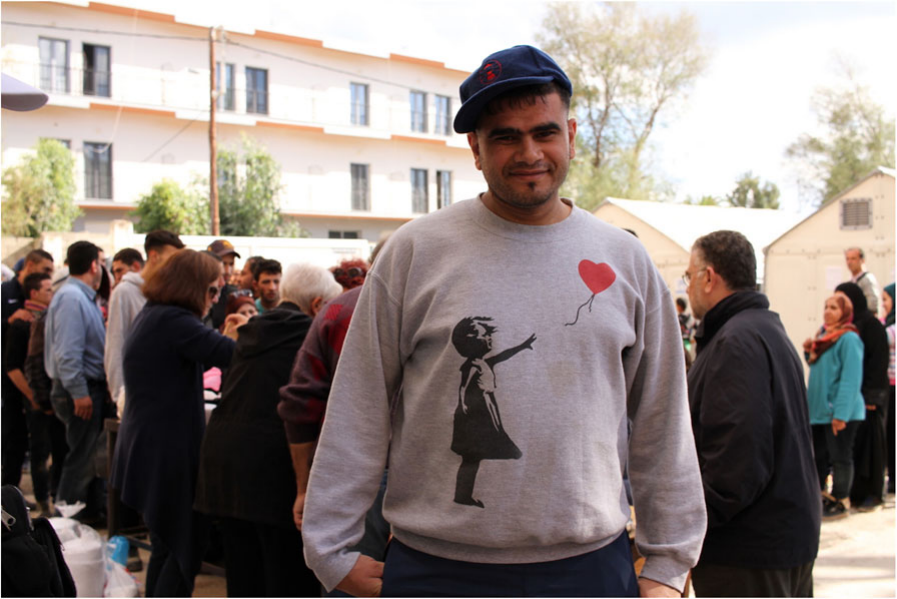
Man posing in the refugee camp during the Sunday meal distribution organized by Leros’ residents. He has just received new clothes, distributed by mutual aid associations. On his jumper, a Bansky drawing. Most smugglers ask migrants to get rid of their luggage before crossing the Aegean Sea so as not to weigh down the inflatable boat. © Laure Gabus

Beyond basic needs, migrants, like all humans, aspire to self-actualisation, have hopes and dreams, and demand dignity (Figs. 2 and 3). They need to be considered as human beings, beyond the stereotypes entertained by certain host populations, and to be freed from any form of discrimination, as indeed assumptions and prejudice inform many political decisions. The humanitarian response in the field also needs to take into account the importance of maintaining communication with those left behind, because of its strong links to psychosocial health and well-being. The first thing that many look for when they have survived a sea-crossing is wireless local area networking (“WiFi”) – to inform and obtain news from loved ones [12]. Social isolation is also a reality, with over two-thirds of migrants stating that needs for social contact were unmet, and this has direct mental health consequences [13, 14].

### Universal health coverage

First recognised by the UN in 2010 [15], the commitment to universal health coverage was subsequently reinforced [16] and is a core component of sustainable development at the global level [17–19]. It requires that all people have access to health services – including prevention, treatment, rehabilitation and palliative care – without risk of financial hardship [20]. Its impact on migrants’ health would be even more positive if this policy were embedded into a broader perspective of universal social rights coverage as put forward by the International Convention on the Protection of the Rights of All Migrant Workers and Members of Their Families adopted by United Nations General Assembly resolution 45/158 of 18 December 1990.

For universal health coverage to occur, research and public health action must take place all along the migratory route, not just upon arrival in a host country. The health status and challenges of many migrating populations are insufficiently addressed until they arrive in a high-profile country. There is, therefore, a clear need to provide and improve healthcare along migration routes. This requires the alignment of the public health and humanitarian agendas, all the more so because health and social systems along the way are often weak.

### Vulnerability

The unprecedented number of forcibly displaced migrants is compounded by increasing demographic, socio-economic, and medical complexity. For example, the global number of child refugees has reached alarming levels: at least 300,000 unaccompanied children moving across borders were registered over two years in 2015–2016, representing a nearly five-fold increase from 2010 to 2011 [21]. Proportions of pregnant women, the elderly and people with disabilities halve also increased in the past years. Migrants are more than ever a heterogeneous group, migrants in irregular status can often be averse to sharing personal details with any administration – including hospitals or medical services – out of fear and/or distrust. Their motivations or even their desired destination may change during the journey. This unprecedented diversity leads to an extended range of medical requirements [22, 23] as well as complex gender and social issues [24–26].

The “healthy migrant” hypothesis suggests that self-selection prior to migration leads to the observation that upon arrival, migrants tend to be younger and fitter than host (or origin) populations [27]. Emerging knowledge on the evolution of newcomers’ health shows that this effect subsides over time, and that migrant health deteriorates after several years due to poverty, poor living conditions, and restricted access to healthcare [27, 28]. The healthy migrant effect may therefore be called into question, as has been suggested by several authors [27, 29, 30].

Violence is a key risk factor for forcibly displaced migrants. In studies in Médecins Sans Frontières (MSF) mental health clinics in Serbia, up to a third of migrants were found to have been victims of violent events [31]. Potentially traumatic events were experienced by 60% and 90% of migrants in their home country and during migration, respectively [32]. In Morocco, among 154 sub-Saharan migrants, 90% reported cases of multiple victimizations, 45% of which were sexual, predominantly gang rape; 79 respondents (51%) were personally victimized, and 27% were forced to witness relatives or co-migrants being victimized [33]. Prisons in all countries are prone to violence, and some administrative detention centres can be even more violent than civilian prisons, due to the absence of rights traditionally granted to prisoners [34], and failiure to follow the UN Standard Minimum Rules for the Treatment of Prisoners (“the Nelson Mandela” rules) [35]. The proportions of foreign inmates in prisons can be very high (e.g. 72% in Switzerland); some are undocumented migrants in administrative detention [36, 37]. Also, due to overcrowding, these are settings where the transmission of infectious diseases of public health interest can occur, especially tuberculosis and sexually transmitted diseases such as syphilis, HIV and hepatitis [38, 39].

In practice, clustering often occurs in migrant populations, with several diseases or conditions affecting the same individuals or groups. This is due to shared vulnerabilities, lack of financial resources, length and duration of the journey and many intermediate destinations, in addition to the epidemiological burden in the country of origin. There are specific risks for women, children (especially unaccompanied minors) and the elderly. Overcrowding and deficient water and sanitation in camps and reception facilities increase risks related to infectious diseases, e.g. vaccine-preventable diseases. Thereafter, restrictive policies in the destination country affect living conditions by limiting access and accessibility to healthcare, education, labour market as well as increasing language and other communication barriers [40].

**Fig. 4:**
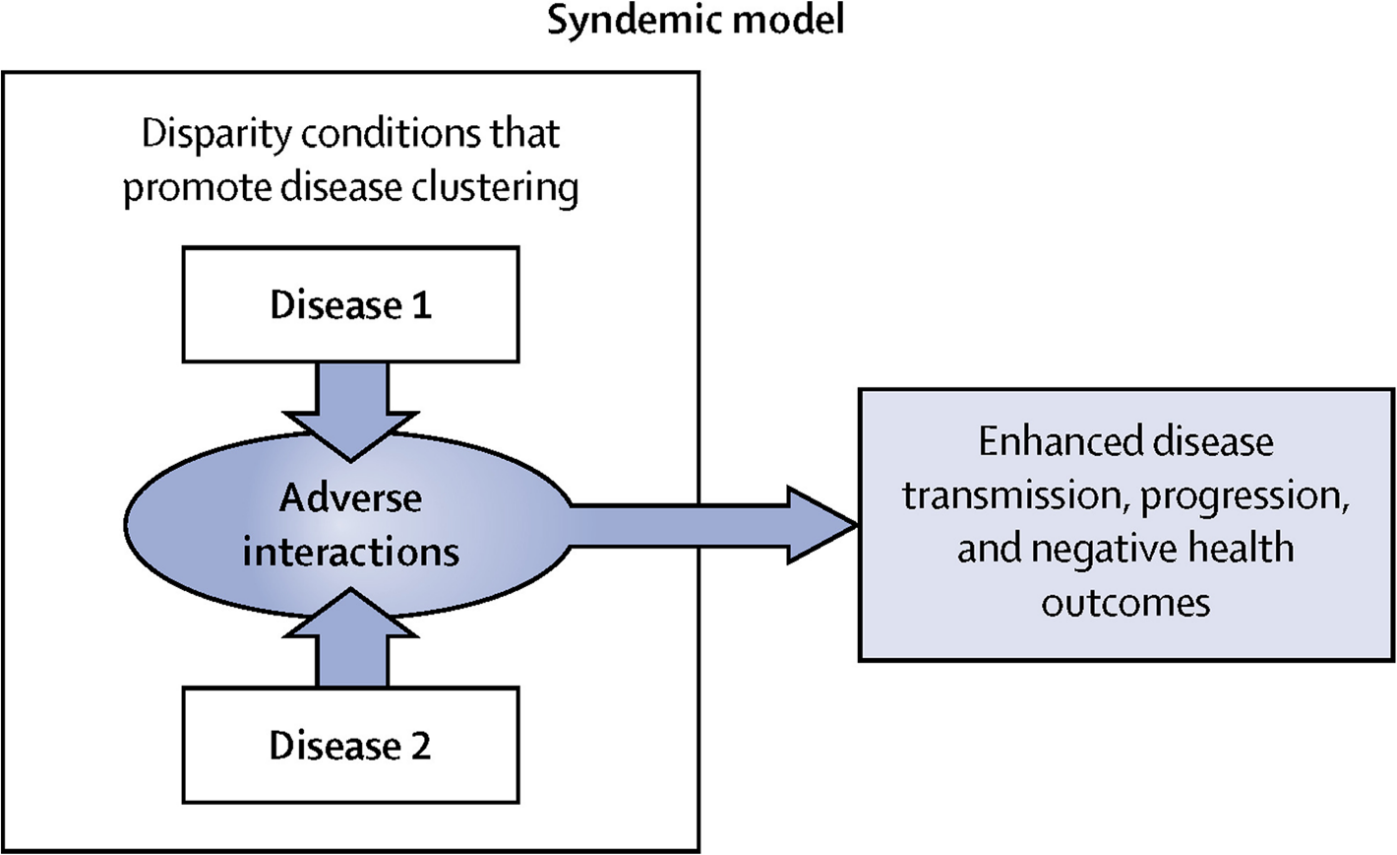
The syndemic model. Reproduced with permission from [41]

The concept of syndemics (synergistic epidemics) can be useful to approach the clustering of certain risk factors or diseases, in certain populations and settings [41]. For example, in a context of migratory stress, synergy between infectious diseases, metabolic diseases and mental health would yield a worse outcome (Fig. 4). Syndemics such as SAVA (substance abuse, violence and AIDS) or VIDDA (violence, immigration, diabetes, depression and abuse) serve as pertinent examples [41].

### Medical footprint and burden of disease

The *medical footprint* is a useful framework to understand each migrant’s personal health capital and its evolution. Whenever a person decides to move, they bring with them a social, cultural and economic capital, which is liable to change during the trajectory of the individual through time and space. Each migrant also has a *personal health capital*, which will also evolve during their journey from their home country to an eventual destination. It is important to take into account migrants’ health capital, and its evolution, the latter being impacted by social determinants of health, and advocate for enabling polices to maintain and develop it. This is important for health equity – universal coverage cannot be realised if certain populations are left aside – but also because there are interactions between migrant and host populations. This dynamic sequence of events can be divided into six stages, each characterised by shortages and medical implications (Table 1).

**Table 1:** Key steps and health determinants of migrants’ health – medical footprint

Step	Main problems/issues	Shortages
1. Pre-migration health experience	Local epidemiological situation and poverty, conflict and war	Diagnosis, vaccination, healthcare, clean water, adequate housing, personal safety
2. Transit health experience	Long in time and space, often worse than in country of origin	Water, nutrition, hygiene, sanitation, housing (overcrowding), social and sexual protection (hostility of resident populations, exploitation by criminal gangs). Exposure to new pathogens for which they have no immunity
3. Destination experience	Unfavourable and unhealthy. Lasting situations governed by the will to survive. Adverse weather conditions outdoors, or if indoors overcrowded conditions and risk of transmission of infectious diseases among migrants	Lack of appropriate clothes, shoes and personal belongings (often abandoned, lost or stolen before or during sea crossings), lack of psychosocial support
4, Healthcare access/use experience	Fear of the law, suspicion of giving out personal data and the general feeling of not being appreciated may affect the evaluation by migrants of their right to access healthcare and other services	Trained healthcare personnel
5. New transit experience	There are often several transit experiences, for instance through North Africa and Southern Europe; through Turkey and the Balkans; or through Central America and Mexico	Water, nutrition, hygiene, sanitation, housing (overcrowding), social and sexual protection (hostility of resident populations, exploitation by criminal gangs). Exposure to new pathogens for which they have no immunity
6. Final destination experience	If and when a migrant finds a job, it is often dirty, dangerous and degrading (“3 Ds”). It may also be illegal, with no insurance coverage and limited access to healthcare. These informal jobs are vitally important for the economies of high-income countries	Lack of appropriate clothes, shoes and personal belongings (often abandoned, lost or stolen before or during sea crossings), lack of psychosocial support

Even if, and it is hardly ever the case, healthcare may be available and relatively accessible when migrants arrive in a host country, this rarely compensates the months and years spent in either a risk-ridden and often prolonged transit phase, or at the point of origin [42]. For migrants, there usually is a succession of stressful incidents or phases: the experience of exile itself, followed by fear linked to life-threatening situations such as crossing the Mediterranean on an overcharged boat, and then administrative anguish as they wait for applications to be processed in a camp or underground shelter. A further stressor might be the uncertainty of their future in a potential host country. Moreover, exposure to conflict and war has a lasting impact on mental health. Due to these life events, the prevalence of psychiatric disorders in refugee populations is much higher than in those not forcibly displaced [14, 43–45]. The link between environmental and psychological stress and adverse health outcomes is well documented. Mental health conditions including anxiety and depression are associated with certain infectious diseases, as has been suggested in a recent review [46].

Forcibly displaced populations are increasingly facing the triple burden of chronic non-communicable diseases (e.g. diabetes, cardiovascular diseases, respiratory conditions and cancer), infectious diseases (e.g. tuberculosis, HIV, hepatitis), and psychiatric illnesses (e.g. post-traumatic stress disorder, depression). A recent survey of a Jordanian camp mainly populated by Syrian refugees indicated prevalence rates for hypertension of 21% and 52%, cardiovascular disease of 7.5% and 21%, diabetes of 12% and 32%, in the 40–59 and 60+ age groups, respectively [47]. Due to the increasing complexity and diversity of migrant populations, there is also the problem of overlapping medical conditions further perplexing appropriate interventions. Increasingly, polymorbidity, often arising at a premature life stage, becomes a challenge among forced migrants in Europe [48]. The impact of community-acquired infections leading to admission to intensive care units seems much higher in refugees in host countries than in the autochthonous population [49, 50].

### Infectious diseases

The prevalence of certain chronic parasitic diseases in asymptomatic migrants reflects, in general, the epidemiologic burden at the country of origin and may be high, up to 5.8%, 48.5%, and 56.1%, for schistosomiasis, Chagas disease, and strongyloidiasis respectively, according to one report [51]. Knowledge of such “tropical diseases”, which may be a misnomer as they have become global, is imperative for proper patient management. Furthermore, more efforts should be undertaken to include migrants in programmes aiming to eradicate neglected tropical diseases [52]. The prevalence of chronic viral diseases, such as HIV, hepatitis C (HCV), and hepatitis B is also higher than in host populations and can be as high as 2.3%, 1.3%, and 14%, respectively, depending on country of origin [51]. Co-infections, e.g. between viral and parasitic infections increase susceptibility to infection, risk of transmission, as well as severity and progression of the disease [53–56]. This may be particularly concerning for chronic viral infections that lead to cancer, such as chronic hepatitis associated hepatocellular carcinoma, or human papillomavirus associated cervical precancerous and cancerous lesions [57]. Migration into Europe is changing the epidemiology of many diseases, including HCV. If the goal of HCV eradication is to be achieved, more inclusive policies and practices will be required [58].

Epidemiological screening is a legitimate tool to better study the profile of migrant populations and to understand their needs [59, 60]. In developing countries facing high rates of internal displacements, there is an urgent need to unify screenings and treatments, and to fight the as yet largely unaddressed problem of counterfeit medicines. In the more developed destination countries, screening of migrant populations seeking healthcare may also help to understand and control the inter-country spread of antibiotic resistance [61]. Health assessment on arrival is a useful way of gaining an initial understanding of the health of incident migrant populations. Screening can be systematic or performed on a case-to-case basis, whereupon arrival, each migrant obtains a personal consultation to describe their medical and transit history [59, 60]. Based on this information, the physician can decide whether to test the person for an array of medical conditions. One such EU-level initiative is the development of the electronic personal and health record, Re-Health (http://re-health.eea.iom.int/). A recent systematic review has estimated that approximately 3% of screened individuals have an infectious disease, but 15% have latent tuberculosis [62]. Furthermore, it was shown that not only is uptake of screening high by migrant populations, but that screening is an effective strategy with moderate/high cost-effectiveness [62]. Evidence also shows positive cost-effectiveness and public health effects of screening and providing early treatment to Latin American women of child-bearing age at risk of suffering and transmitting Chagas disease outside endemic countries [63, 64].

### Vaccine-preventable diseases and vaccination

Screening can also include assessment of immunity to vaccine-preventable diseases. Several studies have shown that vaccination coverage of migrants on arrival is insufficient, although heterogeneity exists between different countries of origin [65–69]. A recent study in Denmark has shown that one third of asylum-seeking children were not immunised in accordance with the national guidelines [65]. This incomplete immunisation may have consequences in terms of outbreaks in refugee camps [70–72].

The WHO-UNHCR-UNICEF Joint Technical Guidance recommends that migrants should be immunized according to the immunisation schedule of the country in which they intend to stay for more than 1 week [73]. It also states that access should be “non-discriminatory and equitable”, and that measles-mumps-rubella (MMR) and polio vaccines should be a priority [73]. A similar strategy is also endorsed by the ECDC [74]. Unfortunately, many countries in Europe have yet to put in place directives on immunisation of migrants [75]. Implementing this strategy, however, is not without challenges related to the migrant condition (lack of information on immunization status, high mobility of migrants, economic difficulties), and further efforts are required in order to harmonise practices and improve communication between host countries and/or agencies [69, 76].

Benefits of achieving adequate vaccine coverage include a decrease in the burden of infectious diseases, and prevention and/or termination of outbreaks; as such, there has recently been interest in the effects of vaccination in reducing AMR [77–80]. It has been previously shown that vaccination strategies against *Haemophilus influenzae* and *Streptococcus pneumoniae* have been associated with decreases in the incidence of infections with resistant pathogens [77–80]. Presumably, one of the several proposed mechanisms would be mediated by decreases in the overall incidence of disease, including that of resistant strains as well as decreases in transmission of antibiotic resistant strains, although further research is required in order to fully understand and develop models [80, 81].

### Health risks: For us or for them?

It has been known for many years that human mobility is linked to transmission, but also susceptibility, to infectious diseases. Movement of people, animals and goods has allowed dissemination of infectious diseases at least since 1000 B.C. [82]. Often, the health risk for the host populations has been politicised by various political groups with an anti-immigration agenda (e.g. Front National in France, Alternative für Deutschland in Germany, Schweizerische Volkspartei in Switzerland) to create a climate of fear surrounding migration. To counter this, the European Centre for Disease Control and Prevention (ECDC) produced a technical document which states that newly-arrived migrants and refugees “do not represent a significant risk for EU/EEA populations” with regards to communicable diseases [83]. This document also states that the risk to refugees has increased due to overcrowding at reception facilities, with the potential for increased transmission of entities like meningococcal disease, measles, varicella and influenza.

Antimicrobial resistance may be considered an emerging infectious disease, and is clearly linked to human mobility [84, 85]. Prevalence of carriage of methicillin-resistant *Staphylococcus aureus* and multi-drug resistant *Enterobacteriaceae* in migrant populations may be as high as 27% [61, 85–87]. Migrants are also overrepresented in terms of multi-drug resistant tuberculosis compared to host populations [88]. A recent systematic review suggests that migrants might acquire antimicrobial-resistant pathogens during the migration process or once they have arrived in the host country; indeed, the prevalence of AMR in the latter would be a major determining factor in transmission to migrants [87]. This may have implications for infection control policies if migrants are hospitalised in low-endemicity settings, as is the case for returned travellers and repatriated patients. Epidemiological screening may be a legitimate tool to better study the profile of migrant populations and understand their needs, and may also help understand and control the spread of antibiotic resistance [61]. It should not, however, be used as a political tool.

### Humanitarian response

Non-governmental partners such as MSF or the International Red Cross and Red Crescent Movement, as well as UN agencies such as UNHCR and IOM, are at the forefront of attempts to manage acute and lasting migratory flows, and are confronted with a considerable diversity of profiles and needs [89]. All too often, the humanitarian response is under such financial and time constraints that its only realistic objective is to help individuals and families survive the current trip and arrive at their next destination without an increase in their medical and psychosocial problems. A recurrent concern is that the international media only focus on migrants when they reach via dramatic journey a high-profile, and often high-income country. Obstacles to integration for regularly residing migrants need to be addressed as well, in the interest of public health and social cohesion. Furthermore, the vast majority of migrants are IDPs in Asia or Africa, where resources for vital interventions are often lacking. Proportionately, IDPs pay the highest burden for mortality, morbidity and malnutrition. Along the migratory pathways, there is a need to standardise diagnostic and treatment protocols, particularly because of chronic diseases requiring continuity of care. This has recently been a problem in many settings, from Lebanon to Ukraine.

The humanitarian response has had to handle many challenges that are either invisible or constantly overlooked, such as interpersonal violence or mental and sexual health. Other challenges include the safety of humanitarian and healthcare personnel [90]. If a hospital is attacked, beyond immediate victims, people will stop going there, which will engender more victims [90, 91]. The Health Care in Danger Project (part of the International Red Cross and Red Crescent Movement) has documented 1809 attacks against healthcare providers worldwide in 2012–2013, 40% of which were directed against healthcare facilities [92]. In the report, it was found that State armed and security forces (military and police) and armed non-State actors are equally involved in these attacks. Use of access to healthcare as a military strategy, known as the weaponisation of healthcare, as for example in Syria, is unacceptable [91]. Unfortunately, evidence suggests that these attacks may be increasing with time [90]. Warring parties and governments need to understand, respect and provide the basic provision of sanitation and health. Also, some migrants are not able or willing to wait for help to arrive: when a team comes to examine a group, it may have moved on, as was experienced by MSF in the Balkans in 2015–2016.

### Focus on policy

The health strand of the Migration Integration Policy Index (MIPEX), collaboratively developed by IOM, has 38 indicators for health policy that can be measured and be addressed towards achieving health equity (health being one of eight sectors covered by MIPEX). Health indicators fall into four dimensions: entitlement to health services, policies to facilitate access, responsive health services and measures to achieve change. According to MIPEX, even well-performing host countries such as Germany or Sweden only achieve around 70% health equity [93].

Policies towards migrants in Europe and the United States tend to be volatile and election-dependent. They are also poorly coordinated with each other. A case in point is the so-called “Dublin Treaty” (the Dublin III Regulation Number 604/2013 came into force on 19 July 2013) which makes the first EU Member State where fingerprints are stored or an asylum claim is lodged, responsible for a person’s asylum claim. This is one of the reasons why Italy and Greece have had to deal with so many migrants and have consistently felt let down by the international community. This policy, as well as its underlying assumptions, has been criticised in the past by UNHCR, and the Parliamentary Assembly of the Council of Europe [94–97].

Because of the Dublin Treaty, many migrants are sent back to the first European country where they were registered, which is often Italy or Greece. These countries have limited perspectives for the realisation of their dreams of economic opportunity. Deportation to the first “Dublin” country often leads to depression, suicidal thoughts or risky behaviour such as unprotected sex or substance abuse.

At the UN General Assembly in September 2016, Member States issued the New York Declaration for Refugees and Migrants, which is a set of commitments as well as an action plan to implement these commitments [98]. This has resulted not only in the Comprehensive Refugee Response Framework, the core elements of which have been agreed on, and which contains four key elements (easing pressure on host countries, enhancing refugee self-reliance, expanding third-country solutions, and supporting conditions in countries of origin for return in safety and dignity), but also the Global Compact on Refugees which will be presented by the High Commissioner for Refugees at the General Assembly in September 2018 [99].

## Conclusion and way forward

Humanitarian problems require political solutions, therefore political commitment is sorely needed to try to reduce the number of uprooted people, and improve their conditions when they are on the move. There is a need to work in a concerted manner on points of origin, points of transit and final points of destination.

The improvement of health of populations, as set out in the UN Sustainable Development Goals, which includes for the first time a migration target (Goal 10), requires the medical and scientific community to understand the complex dynamics of migration. A better grasp of the forces involved is necessary, using a trans-disciplinary approach combining humanitarian, economic, sociological and public health approaches. Accessing and improving basic rights including healthcare along transit routes is a definite priority.

Academics also have a responsibility in lending their voice to the cause of bettering the condition of migrants, and indeed many have taken or called for action [100, 101]. Conducting research that sheds light on the plight of migrants, or on how policy can negatively affect their existence is valuable. Improving awareness of primary-care teams to specific migrant health issues as well as transcultural dimensions by training is another such example [102]. One of the aims of the UCL-*Lancet* Commission on Migration and Health, a multidisciplinary group of academics, policymakers, and health system experts, is to “articulate evidence-base approaches to inform public discourse and policy”and will produce a report set to coincide with the UN General Assembly in September 2018 [103].

This article lends support to recent calls for improved governance mechanisms to ensure the integration of migration within health systems, currently designed for resident populations [104]. Whereas there always seem to be sufficient funds for walls, borders or barriers to the movement of people, more investment is necessary to achieve universal health coverage. Research within Germany has shown that regions that invested less in healthcare for migrants have ended up spending more in the long run [105]. Likewise, a review of the resources invested by the UNHCR in 70 sites in 17 countries shows that increased spending on refugee populations is correlated with lower mortality, reflecting not only efficacy on the part of humanitarian action but also the considerable vulnerability and dependence of migrant populations on international aid [106].

Health equity and early access to healthcare appear as critical responses to the migratory crisis. The principles of public health equity mean that medicine must be used to assist human populations in distress. This commitment at a global level must be followed by concerted actions in the field, where migrants need assistance and protection. Too often, they are denied healthcare or health insurance. If universal health coverage is to be achieved, it cannot be conditioned upon the status of any person [107].

Another point is the opportunity to look at the positive aspects of migration. Global remittances from migrants to their countries of origin have been estimated by the World Bank to be $429 billion [108], which is higher than the “net official development assistance (ODA) flows from member countries of the Development Assistance Committee (DAC) of the OECD” of $135.2 billion in 2014 [109]. Also, a majority of adult migrants have skills that could be put to good use [110–112]. Among migrants and refugees are engineers and healthcare professionals who are able and willing to help, but often cannot do this due to administrative hurdles. It makes sense to find ways of employing these professionals, thus breaking their economic dependence, whilst giving them recognition, and increasing cost-efficiency and the overall well-being of both migrant and host populations, not to mention stopping the drain of human resources affecting low-to middle income countries. This was recognized by the EU when it launched the “science4refugees” initiative [113]. Finally, much of the healthcare provided to migrants during the 2015–2017 crisis was by volunteers. These dedicated people need to be supported by sufficiently strong healthcare, administrative and financial systems [114].

In the words of the UN High Commissioner for Refugees, Filippo Grandi, we must “ask ourselves what each of us can do to overcome indifference or fear and to embrace the idea of inclusion, to welcome refugees to our own communities, and counter narratives that would seek to exclude and marginalise refugees and other uprooted people” [115].
